# Phytochemical Analysis, Antioxidant and Anticancer Potential of *Sideritis niveotomentosa*: Endemic Wild Species of Turkey

**DOI:** 10.3390/molecules26092420

**Published:** 2021-04-21

**Authors:** Ela Nur Şimşek Sezer, Tuna Uysal

**Affiliations:** Department of Biology, Faculty of Science, Selçuk University, Konya 42130, Turkey; tuysal@selcuk.edu.tr

**Keywords:** apoptosis, duvaklı çay, RT-PCR, Turkey

## Abstract

*Sideritis niveotomentosa* Hub. -Mor. is a local endemic species belonging to the Lamiaceae family. In this study, GC/MS analysis, total antioxidant capacity and anticancer effects of different extracts obtained from *S. niveotomentosa* were investigated comparatively. Total phenolic contents of extracts were determined by the Folin–Ciocalteu method, total flavonoid contents by aluminum chloride method, and also the free radical scavenging activities of the extracts by DPPH (2,2-diphenyl-1-picryl-hydrazyl-hydrate) assay. The cytotoxic effect of the extracts was studied via MTT (3-(4,5-Dimethylthiazol-2-yl)-2,5-Diphenyltetrazolium Bromide) assay on DLD1, HL60 and ARH77 cell lines. Pro-apoptotic gene expression levels were also tested in the most sensitive cell line ARH77 by Real-Time PCR. The expression levels of 4 pro-apoptotic genes, *APAF*, *BAX*, *CASP3*, and *HRK* were found to be upregulated in ARH77 cells that were treated extracts. Results showed that methanolic extracts contain more phenolic content than acetone extracts, consistent with DPPH results. As a result, *Sideritis niveotomentosa* extracts, especially methanolic extracts, are rich in phenolic content and have a strong radical scavenging effect. In addition, the extracts showed selective effects on cell lines. This study is pioneering in terms of future studies, and the findings provide hope for future experimentation.

## 1. Introduction

Plants are sources of various natural compounds with different chemical structures and a wide variety of biological activities [1]. The therapeutic use of herbs and their extracts dates back to ancient times, and many effective treatments take place in this way [2]. The most active agents that can be administered for specific therapeutic purposes are products of secondary metabolic pathways. A lot of research in the field of biology, chemistry and medicine is directed towards the identification and characterization of plant secondary metabolites with a pharmacological activity that may be candidates for the synthesis of new drugs. Turkey, home to 11,707 plant taxa, including endemic 3649 [3], is a key country for the protection of global biodiversity in Anatolia because of its complex topography, geomorphology, and location. Among the official plants, the Lamiaceae family contains extremely interesting examples offering natural ingredients and high-quality raw materials [4]. The Lamiaceae family consists of approximately 220 genera and 7000 species worldwide [5]. Some of the major genera belonging to the Lamiaceae family are *Salvia, Mentha, Thymus* and *Sideritis*. Many wild species of the Lamiaceae family are of high importance for their essential oils and antioxidant compounds widely used in medicine, cooking and cosmetics [6,7]. The genus *Sideritis*, belonging to the Lamiaceae family, includes herbaceous plants commonly known as “iron grass”. Official species mainly in the Mediterranean, especially the 140 native species that are known from Spain, Italy, Albania, Bulgaria, Greece and Turkey, and roughly 320 subspecies, are divided into ecotypes and varieties [8]. 46 species and 53 taxa of the genus have existed, and 40 of *Sideritis* taxa are endemic in Turkey [3]. It is known that the chemical components responsible for the pharmacological activities of all *Sideritis* species are terpenes, flavonoids and essential oils [9]. Various studies explain the basis of traditional medicinal uses of *Sideritis* species and increase motivation to find new pharmacological effects [10,11,12]. In addition, a number of therapeutically useful compounds have been extracted from *Sideritis* species, and the genus has been shown to be a valuable reservoir of bioactive substances [13,14,15,16,17]. *Sideritis* species have traditionally been used as teas, sweeteners or for therapeutic purposes. Decoction or infusion of aerial parts of the plant applied orally or topically, is used in folk medicine as anti-inflammatory, antimicrobial, anti-ulcerative, antispasmodic, analgesic, anticonvulsant and degassing agents [18]. Uses everywhere are based on plant properties. Turkey’s Taurus Mountains, *Sideritis pisidica,* Boiss & Heldr. The boiled leaves are used to treat abdominal pain; barley flour, prepared with shredded onions and pine tar and plaster is used as a poultice applied on the abdomen [19]. *Sideritis* species of “mountain tea, spring tea” above-ground parts of the plants which are referred to in Turkey and Greece is widely used to prepare herbal medicines and traditional teas. This tea, often served with honey and lemon, is known for its pleasant aroma, special taste and yellowish color. *Sideritis* tea is widely used to relieve common cold symptoms such as fever, flu, sore throat, and bronchitis, as well as a tonic and diuretic against gastrointestinal disorders such as stomach pain, indigestion and bloating [18]. *Sideritis niveotomentosa* Hub.-Mor. is a local endemic species belonging to the genus. Although there are many studies evaluating the antioxidant capacities of *Sideritis* species, it has been observed that studies on phenolic content and biological activity on *S. niveotomentosa* are very limited [20], which is in line with our literature review as observed in other endemic species of the Mediterranean Basin [21,22]. In this study, we aimed to comparatively reveal the antioxidant cytotoxic and apoptosis-regulating effects of extracts prepared with different solvents and plant parts of the Turkish endemic species *S. niveotomentosa*, as well as the GC-MS analysis.

## 2. Results

### 2.1. GC-MS Analysis of Extracts

**Figure 1:**
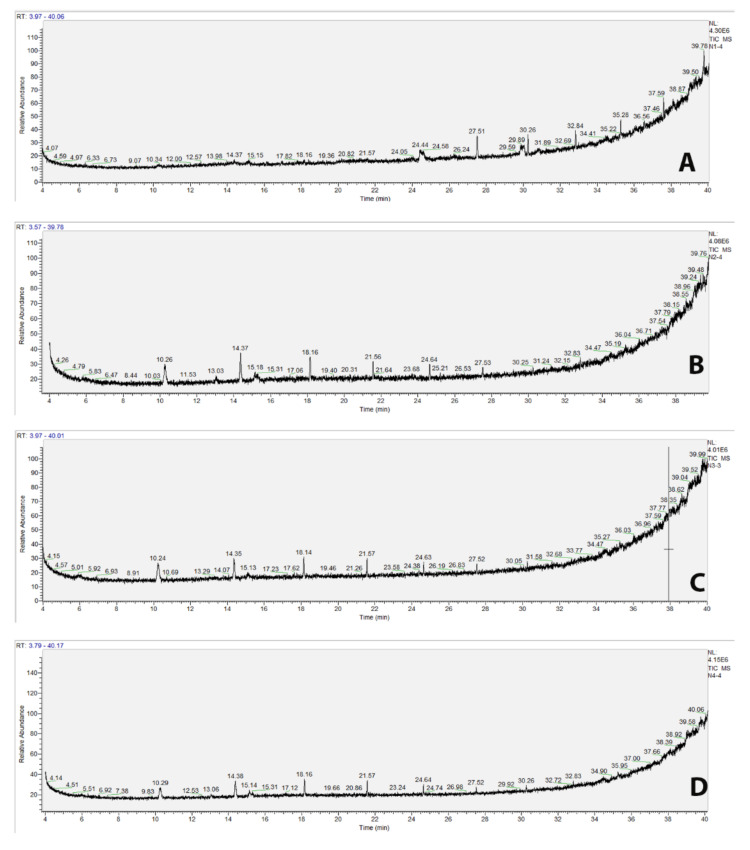
GC/MS chromatogram files of the *S. niveotomentosa* extracts (**A**) methanol leaf extract NTLM, (**B**) acetone leaf extract NTLA, (**C**) methanol flower extract NTFM, (**D**) acetone flower extract NTFA).

The GC-MS chromatogram of methanol and acetone extracts of *S. niveotomentosa* leaf and flower is shown in Figure 1, together with the Retention times (RT). Major plant components in the extracts are presented in Table 1, along with their peak areas.

Totally twenty-six compounds were identified in methanol and acetone extracts of *S. niveotomentosa*. When we look at the content of the extracts, it has been determined that there are some important components with antioxidant and anticancer properties. For example, the antioxidant propyl gallate found in all extracts has been reported to trigger cancer cell death [23]. Another most common component, 1-Monolinoleoylglycerol trimethylsilyl ether has been reported as an antimicrobial, antioxidant and anti-inflammatory agent [24].

### 2.2. Antioxidant Capacity of the Extracts

The extraction yields, TPC, TFC and DPPH IC_50_ values of the extracts are given in Table 2. According to the analysis results, it was determined that methanolic extracts had higher yields. On the other hand, when TPC values were calculated as the equivalent of Gallic acid, it was seen that methanolic extracts had more total phenolic content. When the TFC values were examined, it was determined that the extracts obtained from the leaves contained more flavonoids than the extracts obtained from the flower. Also, it was determined that extracts prepared with methanol contain more flavonoids compared to extracts prepared with acetone.

The radical scavenging activity of the extracts was evaluated by the DPPH assay. According to the results, methanolic extracts show a low IC_50_ value associated with high antioxidant capacity. DPPH results also correlated with the total phenolic content of the extracts.

### 2.3. Cytotoxicity of the Extracts

**Figure 2:**
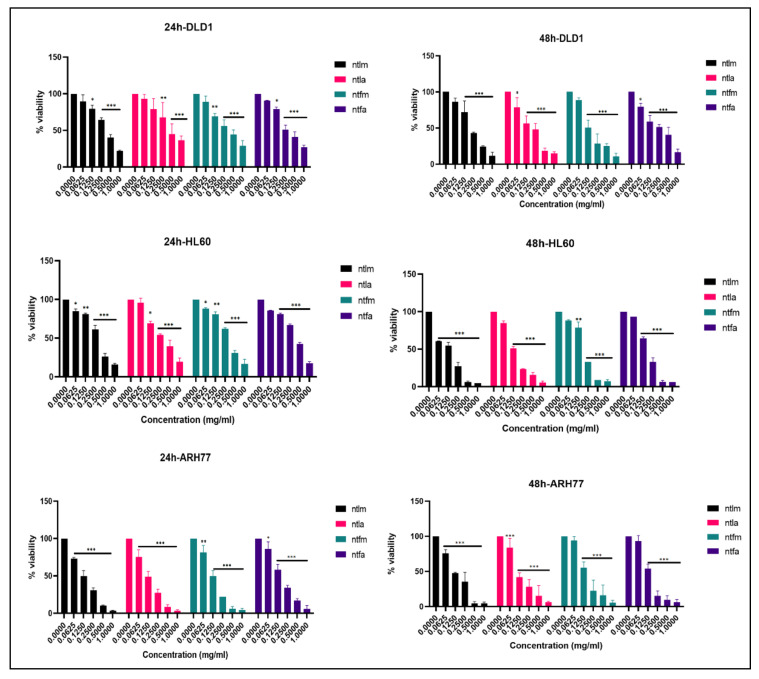
MTT assay graphs of *S. niveotomentosa* extracts (* *p* ≤ 0.05, ** *p* ≤ 0.01, *** *p* ≤ 0.001). (Methanol leaf extract NTLM; acetone leaf extract NTLA; methanol flower extract NTFM and acetone flower extract NTFA).

In this study, cytotoxic activities of methanol and acetone extracts of *S. niveotomentosa* leaves and flowers were tested against DLD1, HL60 and ARH77 cell lines over a 24–48-h incubation period. A wide variety of extract concentrations ranging from 0.0625 to 1 mg mL^−1^ have been used. MTT assay was used for the determination of the cytotoxic activities of the extracts. The data revealed that both extracts had cytotoxic activity against all applied cancer cell types in a dose and time-dependent manner (Figure 2). The extracts applied showed variable effects on different cell lines. Considering the 24 and 48-h IC_50_ concentrations of the extracts, we can say that the lowest IC_50_ concentrations are in the ARH77 cell line and all of the extracts have a strong cytotoxic effect against this cell line. Leaf extracts of *S. niveotomentosa* were found to be more effective in the HL60 cell line at both time intervals. According to the literature, this effect may be due to the flavonoid content of the leaf extracts [25,26,27,28], but additional studies are required to fully establish the cause of the effect.

### 2.4. Real Time PCR Results

In our study, expression levels of four apoptotic gene regions in total were evaluated by the Real-Time PCR technique on the ARH77 cell line. In Real-Time PCR studies, the B-actin was used as a housekeeping gene. Graphs were created using the obtained relative expression levels. When we evaluated Real-Time PCR results in general, it was seen that extracts have different effects on different gene regions and cell death occurred by apoptosis.

**Figure 3:**
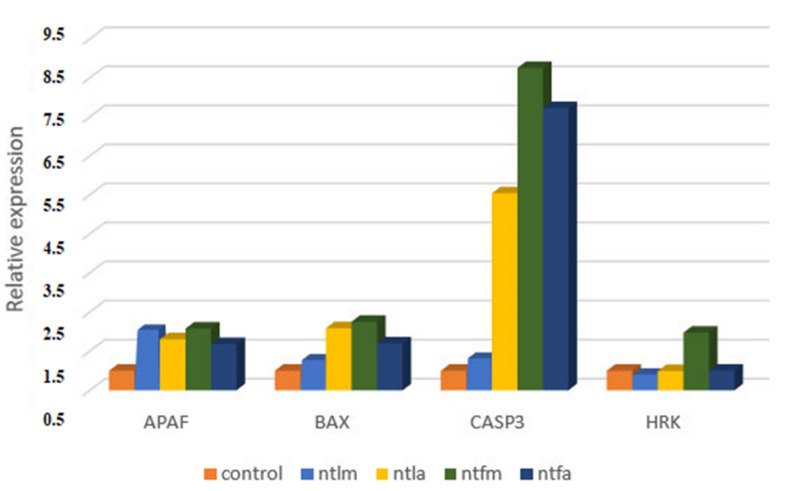
Real-Time PCR results of pro-apoptotic gene regions as relative expression fold change (methanol leaf extract NTLM; acetone leaf extract NTLA; methanol flower extract NTFM and acetone flower extract NTFA).

First of all, when we evaluated the mRNA expression level of *APAF*, we could say that especially methanolic extracts (leaves and flowers) upregulate gene expression (fold change 2 and 2.1). When *BAX* expression were evaluated, the highest upregulation was obtained when NTFM extract was applied (fold change 2.3). When *CASPASE3* gene expression was evaluated, it was found that it upregulated with all extract applications and especially extracts obtained from flower parts were more effective in triggering gene expression (fold change 8.8 and 7.7). The change in *HRK* gene expression was seen only in the group applied with NTFM extract. There was no significant difference in the other groups. The relative expression graphs given in Figure 3.

## 3. Discussion

Since ancient times, people have been using medicinal herbs as alternative medicine. Various teas, extracts and ointments prepared with herbs are preferred as an alternative in the treatment of various diseases, especially cancer.

The species belonging to *Sideritis* taxa used as a traditional herbal tea in Turkey is also used for various medicinal purposes. Different biological activities of *Sideritis* species such as anti-inflammatory, analgesic, antibacterial and antifungal have been reported in previous studies [29,30,31,32,33,34,35,36,37]. According to the GC/MS analyses, in the content of the extracts, it has been determined that there are some important components with antioxidant and anticancer properties. The antioxidant properties of propyl gallate and 1-monolinoleoylglycerol trimethylsilyl ether, which are the most common compounds found in all four extracts, were previously reported [23,24]. Quercetin is a flavonoid that is common in a variety of food and plant types. Quercetin has been shown to inhibit the proliferation of a wide variety of cancers, and recent studies have revealed that quercetin plays an important role as an anti-proliferative and anti-cancer agent, and also stimulates apoptosis [38]. In our study, while quercetin was detected in the extracts obtained from the leaves, it was not detected in the extracts obtained from the flower.

In a study that revealed the antioxidant capacity and diterpenic compounds of the plant, it was reported that methanol extracts had a higher radical scavenging effect than acetone extracts (IC_50 DPPH_: 42.04 ± 0.22 μg/mL and 50.98 ± 0.57 μg/mL respectively) [20]. These data are compatible with our findings. Although the habitat is different, in our study it was revealed that methanol extracts have higher phenolic content and have a stronger radical scavenging potential. When we evaluate the total phenolic amount of *S. niveotomentosa* extracts ranged from 111.84 ± 0.6 to 163.26 ± 0.8 mg Gallic acid equivalents. Methanolic extracts were found to be richer in total phenolic content (TPC), and this finding is consistent with the DPPH radical scavenging activity results obtained. The antioxidant capacity and total phenolic content of previously studied *Sideritis* taxa was consistent with our results [39,40,41].

Plants have been used as an alternative therapy in the treatment of various diseases, especially cancer, for years. Today, cancer is one of the leading causes of death from the disease worldwide. The cytotoxic activities of phytochemicals obtained from plants and plants are particularly important, and more study is required in this area. Various studies have shown that plant extracts, phenolics, flavonoids, and various phytochemicals have potent cytotoxic properties against different types of cancer [42,43]. Based on this, we evaluated the in vitro cytotoxic activity and apoptosis-regulating activity of *S. niveotomentosa* extracts. In this study we evaluate the cytotoxic effects of extracts on three different cell line (DLD1, HL60 and ARH77) and the extracts showed different cytotoxic effects on the cell lines depending on dose and time. The highest cytotoxic activity was detected in the ARH77 cell line at two time intervals. We can clearly say that the cytotoxic effect is cell-specific. According to the literature, different *S. scardica* Griseb extracts exert cytotoxic activity on the rat glioma C6 line and this effect occurs with the arrest of the cell cycle, and the cytotoxic effect has been found to be associated with apoptosis [44]. It was reported that methanol extracts of *S. syriaca* L. significantly affect the proliferation and cell viability of MCF7 cells dose-dependent manner [45]. While the cell lines and species are different, they all point to a consensus that the *Sideritis* species have anticancer potential. The cytotoxic effects of *S. ozturkii* Aytaç & Aksoy leaf and flower extracts on the DLD1 cell line were reported previously and it was determined that especially leaf extract exhibit cytotoxic activity on colorectal cancer cells in a dose and time-dependent [46].

The preferred situation in plant extract and phytochemical applications is that cell death occurs by apoptosis. For this purpose, we evaluated the effects of *S. niveotomentosa* extracts on the expression levels of some pro-apoptotic gene regions in our study. The extracts in our study were found to have positive effects on pro-apoptotic gene expressions. Especially, it was determined that *CASPASE* 3 mRNA expression upregulated significantly among the studied 4 (*APAF*, *BAX*, *CASP3* and *HRK*) gene regions. Caspases are important mediators of programmed cell death (apoptosis). Among them, caspase 3 is a frequently activated death protease that catalyses the specific cleavage of many key cellular proteins [47]. Therefore, upregulation in caspase 3 expression caused by extraction applications may be an indicator of apoptotic cell death. However, additional studies are needed to decide this. In another study, we demonstrated, *S. ozturkii* leaf and flower extracts caused an increase in various apoptotic gene expressions on the DLD1 cell line and especially in caspase 3 expressions [48].

To our knowledge, this is the first effort for the determination of the cytotoxic and apoptosis regulating effects of different *S. niveotomentosa* extracts.

## 4. Materials and Methods

### 4.1. Plant Material

Plant material was collected from its natural habitat at 1295 m from Antalya, Gündoğmuş, Gündoğmuş-Akdağ road. The collection and description of the plant specimen was done by Dr Tuna UYSAL. An herbarium specimen was deposited in Selçuk University KNYA herbarium with collection number TU-3959.

### 4.2. Preparation of Plant Extracts

Firstly, the plant sample was dried without sunlight and prepared for analyses. The leaves and flowers were pulverized under sterile conditions. Subsequently, the prepared 10–20 g of the sample was macerated with acetone and methanol for 2 weeks. The samples were mixed occasionally and stored in the dark. After 2 weeks, obtained extracts were evaporated and yield values were calculated. Extracts were coded as, methanol leaf extract NTLM; acetone leaf extract NTLA and methanol flower extract NTFM and acetone flower extract NTFA, respectively.

### 4.3. GC-MS Analysis

The chemical composition of *S. niveotomentosa* extracts was determined by GC-MS. All analyses were carried out on a Thermo scientific ISQ 7000 Single Quadrupole GC-MS System (Thermo Electron Corporation, USA) with FAME capillary column (30 m × 0.25 mm i.d.; 0.25 μm film thickness). Helium was used as a carrier gas, at a flow rate of 1.0 mL/min. The oven temperature was programmed to increase from 40 to 240 °C at a rate of 5 °C/min, and then held isothermally for 12 min; the total run time was 54 min. Identification was based on the comparison of their RI with those previously reported and by matching their mass spectra with those of Wiley 9 library or literature data. The GC/MS Analysis of the extracts were carried out in ESOGU Research and Development Centre.

### 4.4. Total Phenolic, Flavonoid Content and DPPH Assay

The total phenolic content (TPC) of each extract was evaluated according to the previous method [49,50]. Each extract was prepared at 1 mg mL^−1^ concentration. 3.16 mL of distilled water, 1 mL of methanol and 200 μL of Folin–Ciocalteu reagent were added to 300 μL of this solution taken into a tube. Then, after incubation at room temperature, 600 μL of a sodium carbonate solution was added and the tube was covered with aluminium foil and incubated in a water bath at 40 °C for 30 min. A blank was prepared using the same procedure, but using an equal volume of methanol instead of the plant extract. The absorbance of the extracts was determined at 765 nm. The standard curve of Gallic acid was obtained using the same procedure. The total flavonoid content (TFC) of each extract was evaluated using a previous protocol [51]. In a test tube, first 300 µL of extract, 3.4 mL of methanol (30%), then 150 µL of sodium nitrite solution (0.5 M) and after 150 µL of aluminium chloride solution (0.3 M) were added. After 5-min incubation, 1 mL of sodium hydroxide solution (1 M) was added and the contents mixed well. Afterwards, measurements were made against the blind tube at a wavelength of 506 nm. Results were calculated as rutin equivalents. The radical scavenging activity of the extracts was measured by means of the DPPH test. DPPH analysis was performed according to the Chu method with minor modifications [50,52]. The extracts were added to 0.01% DPPH at various concentrations (0–1 mg/mL) and incubated at room temperature for 30 min. Absorbance was measured at 490 nm and the DPPH radical scavenging activity was calculated as IC_50_ values for each extract.

### 4.5. MTT Assay

In this study, DLD1 (human colorectal cancer), HL60 (human acute promyelocytic leukaemia) and ARH77 (human multiple myeloma) cell lines were used to determine the cytotoxic activity of the extracts. DLD1 and ARH77 cell lines were kindly obtained from Dr Ali Uğur URAL and HL60 cell line from Dr Zerrin CANTÜRK. Cell lines were grown in RPMI-1640 medium supplemented with 10% FBS, 1% Penicillin-Streptomycin and 2 mM L-glutamine, at 37 °C, 5% CO_2_. The prepared extracts were applied to the cell lines at various concentrations (0–1 mg mL^−1^) and time intervals (24–48 h). The cytotoxic potential of the extracts was evaluated via MTT assay. At the end of the incubation period, 5 mg mL^−1^ MTT solution was added to the cells treated with the extracts and left to incubate for 2–4 h. At the end of the period, the contents of the wells were drained and 100 µl of isopropanol was added to each well to dissolve the formazan crystals formed [53]. Plates were read on an ELISA reader at 540 nm wavelength. The effect of the extracts on cell viability was calculated by comparing the absorbance values obtained from the control group (no treatment) Analyses were done in triplicate, at least 2 replicates per plate. Mean values for cell viability values were considered. Statistical analysis was performed using Graph Pad Prism 9 for Windows (Graph Pad Software, San Diego, CA, USA). Data were compared using one-way ANOVA and post hoc Dunnett’s test (* *p* ≤ 0.05, ** *p* ≤ 0.01, *** *p* ≤ 0.001).

### 4.6. Real-Time PCR

Total RNA isolation was performed with the Bio-Rad Aurum Total RNA Isolation kit (Bio-Rad, Hercules, CA, USA) according to the manufacturer’s recommendations, and the quality and concentration values of the total RNA samples were determined with Nanodrop 2000 (Wilmington, DE, USA). 0.5–1 micrograms of total RNA were reverse transcribed into cDNA using the iScript cDNA synthesis kit (Bio-Rad, Hercules, CA, USA). Primers were taken from the human apoptosis primer library HPA-I (Real Time Primers, LLC, Elkins Park, PA, USA). Gene expression experiments were performed in a final mix volume of 10 µL containing 5 µL SYBR green master mix (Bio-Rad, Hercules, CA, USA), 1 µL primer, 3 µL dH_2_O and 1 µL cDNA. The B-actin gene was used as a house-keeping gene. The mRNA expression levels of Β-actin and apoptotic gene regions were measured by Real-Time PCR. The obtained data were analysed by the comparative CT method and the fold change was calculated with 2^−ΔΔCT^.

## 5. Conclusions

In conclusion, plant extracts and phytochemicals can be considered natural substances that can be used in various fields such as medicine and pharmacology. Especially when endemic and wild plants are considered, it is particularly important in terms of being unique to the country and having its own gene source. It can also be informative about the identification of various pharmacological characteristics of endemic plants and their distinction from other wild species. We believe that this study is important as a resource for plant-based anti-cancer compound studies. Among the studies planned to be carried out in the future, the active substance or substances in the extracts will be revealed and it will be determined whether these substances have an effect alone, or synergistically with other substances. Additionally, different apoptotic markers will be used to determine which pathways trigger apoptotic cell death.

## Figures and Tables

**Table 1:** Major plant components in the *S. niveotomentosa* extracts (methanol leaf extract NTLM; acetone leaf extract NTLA; methanol flower extract NTFM and acetone flower extract NTFA).

	% Area			
Compounds	NTLM	NTLA	NTFM	NTFA
Propyl Gallate	6.53	1.26	2.43	4.22
2-Monolinolenin	4.48	-	4.17	
alpha-d-Glucofuranosyl benzenesulfonate	2.17	-	-	-
1H-Purin-6-amine, [(2-fluorophenyl) methyl]	10.80	-	-	-
5,7-Dodecadiyne-1,12-diol	5.89	-	-	-
Cathine	7.42	-	-	2.74
1-Monolinoleoylglycerol trimethylsilyl ether	6.48	3.93	3.67	4.40
Quercetin 7,3′,4′-Trımethoxy	4.71	2.98	-	-
Cyclohexasıloxane, Dodecamethyl	-	14.10	11.89	9.11
Cycloheptasiloxane, tetradecamethyl	-	14.29	10.82	13.90
p-Tolylthiourea	-	3.73		4.51
Benzoic acid, 2,4-bis[(trimethylsilyl)oxy]-,trimethylsilyl ester	-	9.05	7.48	9.08
Bıstrımethylsılyl N-Acetyl Eıcosasphınga-4,11-Dıenıne	14.03	5.89	5.12	6.66
Anhydrorhodovibrin	-	1.66	1.80	-
Rhodovibrin	-	-	4.14	-
4-(4-Chlorophenyl)-2-(cyclopropyl)-6-[4-[bis(4-fluorophenyl) methyl]piperaziny l-1-yl]benzonitrile	-	-	4.40	-
7,12-Dihydro-6,7-bis(4-hydroxyphenyl)-6H- 1,2,4]triazolo[1′,5′:1,2]pyrimido [5,4-c]chromen-2-ol	7.99	4.00	-	3.40
2-(5-(5-[Cyano-(9,9-dimethyl-1,4-dioxa-7-aza-spiro[4.4] non-7-en-8-yl)-methylene]-3,3-dimethylpyrrolidin-2-ylidenemethyl)-3,3-dimethyl-ë1-pyrrolin-5-ylidene methyl-4,4,5-trimethyl-ë1-pyrroline-5-carbonitrile]	2.50	-	-	6.28
4-(4-Chlorophenyl)-2-(cyclopropyl)-6-[4-[bis(4-fluorophenyl) methyl]piperaziny l-1-yl]benzonitrile	2.01	2.67	4.40	2.05
Silane, trimethyl(phenethylthio)	-		3.14	2.41
8,11-Octadecadiynoic acid, methyl ester	-	2.08		-
9-Desoxo-9x-hydroxy-7-ketoingol 3,8,9,12-tetraacetate	-	1.67	2.18	-
3,6-Dimethoxy-2,5-dinitrobenzaldehydeoxime	-	4.22	-	-
2-(*N*-Acetylanilino)-1,3-selenazol-4-ylm ethyl]triphenylphosphonium iodide	-	13.61	-	2.22

**Table 2:** Antioxidant capacity of *S. niveotomentosa* extracts methanol leaf extract NTLM; acetone leaf extract NTLA; methanol flower extract NTFM and acetone flower extract NTFA).

Assay	NTLM	NTLA	NTFM	NTFA
yield of the extracts %	10.02	3.78	10.55	3.94
TPC (µg mL^−1^ GAE)	163.26 ± 0.8	111.84 ± 0.6	161.09 ± 0.3	119.16 ± 0.15
TFC (µg mL^−1^ Rutin)	64.33 ± 0.02	43.59 ± 0.5	28.69 ± 0.4	18.06 ± 0.37
DPPH IC_50_ values (mg mL^−1^)	0.236	0.404	0.277	0.509

## Data Availability

The data presented in this study are available within the article.

## Sample Availability

Samples of the compounds are not available from the authors.
